# Thymidylate synthase as a determinant of pemetrexed sensitivity in non-small cell lung cancer

**DOI:** 10.1038/bjc.2011.129

**Published:** 2011-04-12

**Authors:** K Takezawa, I Okamoto, W Okamoto, M Takeda, K Sakai, S Tsukioka, K Kuwata, H Yamaguchi, K Nishio, K Nakagawa

**Affiliations:** 1 Department of Medical Oncology, Kinki University Faculty of Medicine, 377-2 Ohno-higashi, Osaka-Sayama, Osaka 589-8511, Japan; 2 Department of Genome Biology, Kinki University Faculty of Medicine, 377-2 Ohno-higashi, Osaka-Sayama, Osaka 589-8511, Japan; 3 Tokushima Research Center, Taiho Pharmaceutical Co. Ltd., 224-2 Hiraishi-ebisuno, Kawauchi, Tokushima 771-0194, Japan

**Keywords:** non-small cell lung cancer, thymidylate synthase, pemetrexed, apoptosis, immunohistochemistry

## Abstract

**Background:**

Although a high level of thymidylate synthase (TS) expression in malignant tumours has been suggested to be related to a reduced sensitivity to the antifolate drug pemetrexed, no direct evidence for such an association has been demonstrated in non-small cell lung cancer (NSCLC). We have now investigated the effect of TS overexpression on pemetrexed sensitivity in NSCLC cells.

**Methods:**

We established NSCLC cell lines that stably overexpress TS and examined the effects of such overexpression on the cytotoxicity of pemetrexed both *in vitro* and in xenograft models. We further examined the relation between TS expression in tumour specimens from NSCLC patients and the tumour response to pemetrexed by immunohistochemical analysis.

**Results:**

The sensitivity of NSCLC cells overexpressing TS to the antiproliferative effect of pemetrexed was markedly reduced compared with that of control cells. The inhibition of DNA synthesis and induction of apoptosis by pemetrexed were also greatly attenuated by forced expression of TS. Furthermore, tumours formed by TS-overexpressing NSCLC cells in nude mice were resistant to the growth-inhibitory effect of pemetrexed observed with control tumours. Finally, the level of TS expression in tumours of non-responding patients was significantly higher than that in those of responders, suggestive of an inverse correlation between TS expression and tumour response to pemetrexed.

**Conclusion:**

A high level of TS expression confers a reduced sensitivity to pemetrexed. TS expression is thus a potential predictive marker for response to pemetrexed-based chemotherapy in NSCLC patients.

Lung cancer is the most common cause of cancer-related death worldwide, with non-small cell lung cancer (NSCLC) accounting for ∼75% of all lung cancer cases (Hoffman *et al*, 2000). Platinum-based chemotherapy is the standard first-line treatment for individuals with advanced NSCLC, but the efficacy of such agents with regard to improving clinical outcome is limited (Schiller *et al*, 2002). Both experimental and clinical studies have revealed that many molecules contribute to the biological activities of malignant tumours including NSCLC. New strategies based on a better understanding of tumour biology may thus help to maximise the efficacy of current treatments.

A relatively new antifolate drug, pemetrexed, inhibits the growth of a variety of tumour types by targeting multiple folate-dependent enzymes including thymidylate synthase (TS), dihydrofolate reductase, and glycinamide ribonucleotide formyltransferase (Shih *et al*, 1997). The antitumour efficacy of pemetrexed has been found to be more limited in lung cancer patients with squamous cell carcinoma than in those with other histotypes of NSCLC (Scagliotti *et al*, 2008). Furthermore, the abundance of TS mRNA or protein seems to be higher in squamous cell carcinoma than in other histotypes of NSCLC (Ceppi *et al*, 2006; Monica *et al*, 2009; Takezawa *et al*, 2010), and high levels of TS expression in various tumour types have been suggested to correlate with a poor response to TS-targeted agents (Johnston *et al*, 1994, 1997; Pestalozzi *et al*, 1997; Ferguson *et al*, 1999). The poorer response of NSCLC patients with squamous cell carcinoma to pemetrexed is thus thought to result from the higher level of TS expression in such tumours. However, such a relation between a high TS expression level and a reduced sensitivity to pemetrexed in NSCLC has not been well established. Moreover, the precise mechanism that might underlie a reduced sensitivity to pemetrexed in tumours with a high level of TS expression remains unknown.

We have now constructed an expression vector for TS and have used this vector to establish several NSCLC cell lines that stably overexpress TS. With the use of these cells, we examined the relation between the anticancer effects of pemetrexed both *in vitro* and *in vivo* and the expression level of TS. We further investigated the relation between pemetrexed sensitivity and TS expression level in primary lung cancer patients.

## Materials and methods

### Cell culture and reagents

The human lung cancer cell lines A549, H1299, and PC9 were obtained from American Type Culture Collection (Manassas, VA, USA). All cells were cultured in RPMI 1640 medium (Sigma, St Louis, MO, USA) supplemented with 10% fetal bovine serum and 1% penicillin-streptomycin (Sigma), and they were maintained under a humidified atmosphere of 5% CO_2_ at 37°C. Pemetrexed, cisplatin, and docetaxel were obtained from Wako (Osaka, Japan).

### Generation of TS-overexpressing NSCLC cell lines

A full-length cDNA fragment encoding TS was obtained from PC9 cells by reverse transcription and the polymerase chain reaction with the primers TS-F (5′-AAGCTTCGCGCCATGCCTGTGGCCGGCTCGGAG-3′) and TS-R (5′-GCGGCCGCCTAAACAGCCATTTCCATTTTAATAG-3′). The amplification product was verified by sequencing after its cloning into the pCR-Blunt II-TOPO vector (Invitrogen, Carlsbad, CA, USA). The TS cDNA was excised from pCR-Blunt II-TOPO and transferred to the pMZs retroviral vector (Cell Biolabs, San Diego, CA, USA). The resulting pMZs construct and the pVSV-G vector (Clontech, Palo Alto, CA, USA) for construction of the viral envelope were introduced into GP2-293 cells (∼80% confluence in a 10-cm dish) with the use of the FuGENE6 transfection reagent. After 48 h, the viral particles released into the culture medium were concentrated by centrifugation at 15 000 × **g** for 3 h at 4°C. The resulting pellet was then suspended in fresh RPMI 1640 medium and used to infect A549, H1299, or PC9 cells as previously described (Okamoto *et al*, 2010).

### Immunoblot analysis

Cells were washed twice with ice-cold phosphate-buffered saline (PBS) and then lysed in a solution containing 20 mM Tris-HCl (pH 7.5), 150 mM NaCl, 1 mM EDTA, 1% Triton X-100, 2.5 mM sodium pyrophosphate, 1 mM phenylmethylsulfonyl fluoride, and leupeptin (1 *μ*g ml^−1^). The protein concentration of cell lysates was determined with the Bradford reagent (Bio-Rad, Hercules, CA, USA), and equal amounts of lysate protein were subjected to SDS-polyacrylamide gel electrophoresis on a 7.5 or 12% gel. The separated proteins were transferred to a nitrocellulose membrane, which was then exposed to 5% non-fat dried milk in PBS for 1 h at room temperature before incubation overnight at 4°C with primary antibodies. Rabbit polyclonal antibodies to human TS were obtained from Santa Cruz Biotechnology (Santa Cruz, CA, USA), and those to *β*-actin were from Sigma. The membrane was then washed with PBS containing 0.05% Tween 20 before incubation for 1 h at room temperature with horseradish peroxidase-conjugated goat antibodies to rabbit immunoglobulin G (Sigma). Immune complexes were finally detected with chemiluminescence reagents (GE Healthcare, Little Chalfont, UK).

### Assay of TS activity

Thymidylate synthase activity was quantified with the use of a tritiated 5-fluoro-dUMP binding assay. Cells were harvested, diluted in 0.2 M Tris-HCl (pH 7.4) containing 20 mM 2-mercaptoethanol, 15 mM CMP, and 100 mM NaF, and disrupted by ultrasonic treatment. The cell lysate was centrifuged at 1600 × **g** for 15 min at 4°C, and the resulting supernatant was centrifuged at 105 000 × **g** for 1 h at 4°C. A portion (50 *μ*l) of the final supernatant was mixed consecutively with 50 *μ*l of Buffer A (600 mM NH_4_HCO_3_ buffer (pH 8.0), 100 mM 2-mercaptoethanol, 100 mM NaF, 15 mM CMP) and with 50 *μ*l of (6-^3^H)5-fluoro-dUMP (7.8 pmol) plus 25 *μ*l of cofactor solution (50 mM potassium phosphate buffer (pH 7.4), 20 mM 2-mercaptoethanol, 100 mM NaF, 15 mM CMP, 2% bovine serum albumin, 2 mM tetrahydrofolic acid, 16 mM sodium ascorbate, 9 mM formaldehyde). The resulting mixture was incubated at 30°C for 20 min, after which the reaction was terminated by the addition of 100 *μ*l of 2% bovine serum albumin and 275 *μ*l of 1 M HClO_4_ and by centrifugation at 1600 × **g** for 15 min at 4°C. The resulting precipitate was suspended in 2 ml of 0.5 M HClO_4_, and the mixture was subjected to ultrasonic treatment followed by centrifugation at 1600 × **g** for 15 min at 4°C. The final precipitate was solubilised with 0.5 ml of 98% formic acid, mixed with 10 ml of ACS II scintillation fluid, and assayed for radioactivity. Data are expressed as picomoles of substrate consumed per milligram of soluble protein.

### Cell growth inhibition assay *in vitro* (MTT assay)

Cells were plated in 96-well flat-bottomed plates and cultured for 24 h before exposure to various concentrations of drugs for 72 h. TetraColor One (5 mM tetrazolium monosodium salt and 0.2 mM 1-methoxy-5-methyl phenazinium methylsulfate; Seikagaku, Tokyo, Japan) was then added to each well, and the cells were incubated for 3 h at 37°C before measurement of absorbance at 490 nm with a Multiskan Spectrum instrument (Thermo Labsystems, Boston, MA, USA).

### RNA interference

Cells were plated at 50–60% confluence in six-well plates or 25-cm^2^ flasks and then incubated for 24 h before transient transfection for the indicated times with small interfering RNAs (siRNAs) mixed with the Lipofectamine reagent (Invitrogen). An siRNA specific for human TS mRNA (5′-CAAUCCGCAUCCAACUAUU-3′) and a nonspecific siRNA (5′-GUUGAGAGAUAUUAGAGUU-3′) was obtained from Nippon EGT (Toyama, Japan).

### Assay of DNA synthesis

DNA synthesis was measured with the use of a Cell Proliferation ELISA BrdU Kit (Roche, Basel, Switzerland). In brief, cells were seeded in 96-well plates at a density of 10 000–20 000 per well and exposed to various concentrations of drugs for 48 h. They were then incubated in the additional presence of bromodeoxyuridine (BrdU) for 3 h before exposure to detection reagents for 15 min at 25°C and measurement of luminescence.

### Annexin V binding assay

Binding of annexin V to cells was measured with the use of an Annexin-V-FLUOS Staining Kit (Roche). Cells were harvested by exposure to trypsin–EDTA, washed with PBS, and centrifuged at 200 × **g** for 5 min. The cell pellets were resuspended in 100 *μ*l of Annexin-V-FLUOS labelling solution, incubated for 10–15 min at 15 to 25°C, and then analysed for fluorescence with a flow cytometer (FACS Calibur) (Becton Dickinson, San Jose, CA, USA) and Cell Quest software (Becton Dickinson).

### Animals

Male athymic nude mice were maintained on a 12-h light, 12-h dark cycle and provided with food and water ad libitum in a barrier facility. All animal experiments were carried out with approval of the institutional animal care and use committee and complied with the specifications of the Association for Assessment and Accreditation of Laboratory Animal Care of Japan.

### Tumour growth inhibition assay *in vivo*

Cubic fragments of tumour tissue (∼2 by 2 by 2 mm) were implanted subcutaneously into the axilla of 5- to 6-week-old male athymic nude mice. Treatment was initiated when tumours in each group of eight mice achieved an average volume of 150–200 mm^3^. Pemetrexed (100 mg per kilogram of body weight) or vehicle (physiological saline) was administered intraperitoneally once a week. Tumour volume was determined from caliper measurements of tumour length (*L*) and width (*W*) according to the formula *LW*^2^/2. Both tumour size and body weight were measured twice per week.

### Patients and clinical specimens

For retrospective analysis, we recruited consecutive patients with advanced NSCLC who received chemotherapy at Kinki University Hospital between April 2008 and June 2010. Patients met all of the following criteria: a histological diagnosis of NSCLC with at least one measurable lesion; a clinical stage of 3B or 4; an Eastern Cooperative Oncology Group (ECOG) performance status of 0 or 1; adequate haematologic, hepatic, and renal function; treatment either with carboplatin at an area under the curve (AUC) of 5 on day 1 and pemetrexed at 500 mg m^−2^ on day 1 of an 21-day cycle or with cisplatin at 75 mg m^−2^ on day 1 and pemetrexed at 500 mg m^−2^ on day 1 of an 21-day cycle as first-line chemotherapy; and availability of sufficient tumour tissue in paraffin blocks for assessment by immunohistochemistry. Tumour tissue specimens were obtained by transbronchial lung biopsy. Tumour response was examined by computed tomography and evaluated according to the Response Evaluation Criteria in Solid tumours (RECIST) as complete response (CR), partial response (PR), stable disease (SD), or progressive disease (PD). This study conforms to the provisions of the Declaration of Helsinki and was approved by the local institutional review board.

### Immunohistochemistry and scoring of TS expression

Paraffin-embedded sections (thickness, 4 *μ*m) of tumour tissue were depleted of paraffin with xylene and then rehydrated, and endogenous peroxidase activity was quenched by incubation with 0.3% hydrogen peroxide in methanol. Antigen retrieval was carried out by microwave irradiation for 10 min in citrate buffer (pH 6.0). The sections were then washed with PBS before incubation overnight at room temperature with rabbit polyclonal antibodies to TS (Taiho Pharmaceutical Co., Saitama, Japan) at a dilution of 1 : 100. Immune complexes were detected by incubation at room temperature for 30 min first with biotinylated goat antibodies to rabbit immunoglobulin G (Dako, Santa Barbara, CA, USA) and then with streptavidin-conjugated horseradish peroxidase (Dako). Peroxidase activity was visualised with diaminobenzidine tetrahydrochloride solution (Dako), and the sections were counterstained with hematoxylin before examination with a microscope (Dako). The human colon cancer cell line DLD-1/FrUrd, human breast cancer cell line MDA-MB-435S, and human pancreatic cancer cell line MIAPaCa-2 (all obtained from American Type Culture Collection) were used as positive controls for TS staining. All immunostained sections were reviewed by two observers without knowledge of the patients' characteristics. Sections with discrepant results were jointly reevaluated until a consensus was reached. Cytoplasmic staining for TS was scored in a semiquantitative manner, reflecting both the intensity of staining and the percentage of cells with staining at each intensity. Staining intensity was classified as 0 (no staining), +1 (weak staining), +2 (distinct staining), or +3 (strong staining). A value designated the HSCORE was obtained as Σ(I × PC), where I and PC represent staining intensity and the percentage of cells that stain at each intensity, respectively. The selection of a clinically important cutoff score for TS expression was based on receiver operating characteristic (ROC) curve analysis.

### Statistical analysis

Quantitative data are presented as means±s.d. or ±s.e.m. as indicated, and were analysed by Student's two-tailed *t*-test. Progression-free survival was assessed from the first day of chemotherapy administration to the date of objective disease progression. Kaplan–Meier analysis was used to estimate the probability of survival as a function of time, and differences in the survival of subgroups of patients were evaluated with the log-rank test. A *P*-value of <0.05 was considered statistically significant.

## Results

### Forced expression of TS reduces the sensitivity of NSCLC cells to pemetrexed

**Figure 1:**
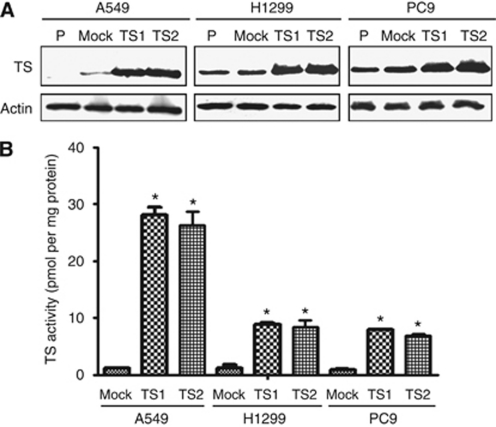
Abundance and enzymatic activity of TS in TS-overexpressing NSCLC cell lines. Parental (P) A549, H1299, or PC9 cells or corresponding sublines either stably overexpressing TS (TS1 and TS2) or harbouring the empty vector (Mock) were cultured overnight in complete medium, after which cell lysates were prepared and either subjected to immunoblot analysis with antibodies to TS and to *β*-actin (loading control) (**A**) or assayed for TS activity. (**B**) Data are means±s.d. of triplicates from experiments that were repeated on two additional occasions with similar results. ^*^P<0.05 *vs* the corresponding value for Mock cells (Student's two-tailed *t*-test).

**Figure 2:**
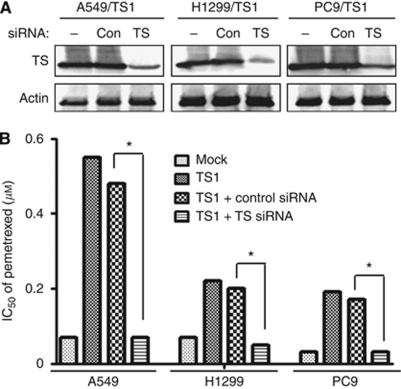
Effect of TS depletion on pemetrexed sensitivity in TS-overexpressing NSCLC cells. (**A**) Cells of the indicated lines were transfected or not (−) with nonspecific (Con) or TS siRNAs for 48 h, after which cell lysates were subjected to immunoblot analysis with antibodies to TS and to *β*-actin. (**B**) Cells transfected as in **A** were cultured for 72 h in complete medium containing various concentrations of pemetrexed, after which cell viability was assessed as described in Materials and methods section, and the median inhibitory concentration (IC_50_) of pemetrexed was determined. Data are means of triplicates from experiments that were repeated on two additional occasions with similar results. ^*^P<0.05 (Student's two-tailed *t*-test).

To investigate whether the level of TS expression affects the sensitivity of NSCLC cells to pemetrexed, we first established A549 (A549/TS1 and A549/TS2), H1299 (H1299/TS1 and H1299/TS2), and PC9 (PC9/TS1 and PC9/TS2) cells that stably overexpress TS. Cells that stably harbour the corresponding empty vector (A549/Mock, H1299/Mock, and PC9/Mock) were established as controls. Immunoblot analysis showed that the abundance of TS was markedly increased in the TS-overexpressing lines compared with the parental or Mock cells (Figure 1A). The enzymatic activity of TS was also substantially higher in the TS-overexpressing cells than in the parental or Mock cells (Figure 1B). We then examined the effect of forced expression of TS on the cytotoxicity of anticancer drugs as determined with the MTT assay. The median inhibitory concentration of pemetrexed for the TS-overexpressing cells was about three to six times that for the corresponding Mock cells for all three lung cancer lines, whereas cisplatin and docetaxel inhibited the growth of the TS-overexpressing cells in a manner similar to that observed with the corresponding Mock cells (Table 1). To exclude the possibility that these results were because of nonspecific effects of transfection, we depleted A549/TS1, H1299/TS1, and PC9/TS1 cells of TS by RNA interference. Immunoblot analysis revealed that transfection of these cells with an siRNA specific for TS mRNA resulted in downregulation of the corresponding protein (Figure 2A). This reduction in the abundance of TS restored the sensitivity of the cells to the inhibitory effect of pemetrexed on cell growth (Figure 2B). These data thus indicated that high TS expression levels reduce the sensitivity of NSCLC cells to pemetrexed.

### Effects of chemotherapeutic agents on DNA synthesis and apoptosis in TS-overexpressing NSCLC cell lines

**Figure 3:**
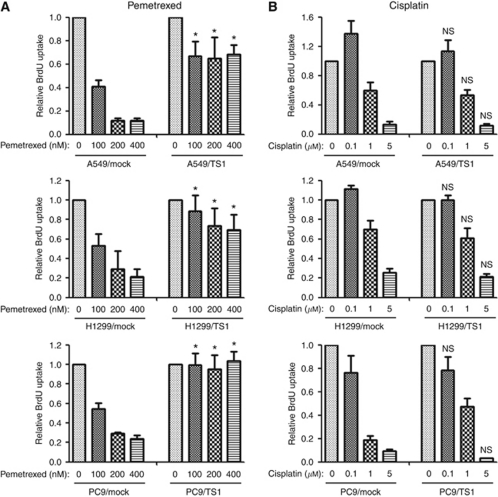
Effects of pemetrexed and cisplatin on DNA synthesis in NSCLC cells overexpressing TS. The indicated NSCLC cell lines were cultured for 48 h in complete medium containing various concentrations of pemetrexed (**A**) or cisplatin (**B**), after which BrdU incorporation was assessed as described in Materials and methods section. Data are means±s.d. of triplicates from experiments that were repeated a total of three times with similar results. ^*^P<0.05 *vs* the corresponding value for Mock cells (Student's two-tailed *t*-test). NS, not significant.

**Figure 4:**
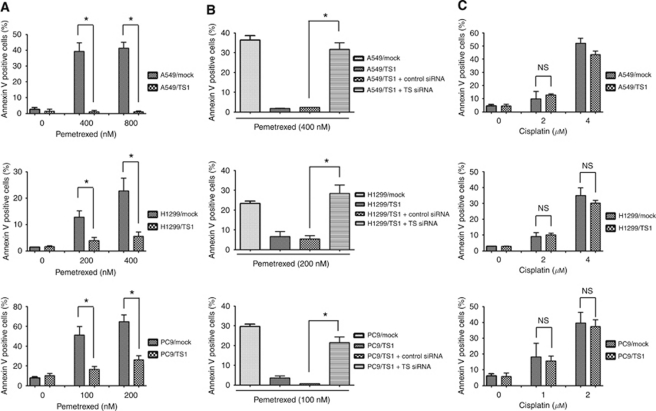
Effects of pemetrexed and cisplatin on apoptosis in NSCLC cells overexpressing TS. (**A** and **C**) The indicated NSCLC cell lines were cultured for 72 h in complete medium containing various concentrations of pemetrexed (**A**) or cisplatin (**C**), after which the proportion of apoptotic cells was assessed by staining with fluorescein isothiocyanate-conjugated annexin V and propidium iodide followed by flow cytometry. (**B**) The indicated NSCLC cell lines were cultured for 72 h in complete medium containing the indicated concentrations of pemetrexed with or without nonspecific (control) or TS siRNAs, after which the proportion of apoptotic cells was assessed as in **A** and **C**. All data are means±s.d. of triplicates from experiments that were repeated a total of three times with similar results. ^*^P<0.05 (Student's two-tailed *t*-test). NS, not significant.

We next investigated the effects of TS overexpression on DNA synthesis and apoptosis in NSCLC cells exposed to pemetrexed, given that the cytotoxic activity of pemetrexed is due to inhibition of DNA synthesis and subsequent induction of apoptosis. Assay of BrdU incorporation revealed that pemetrexed inhibited DNA synthesis in Mock cell lines in a concentration-dependent manner, whereas this effect was much less pronounced in the TS-overexpressing lines (Figure 3A). In contrast, the concentration-dependent inhibition of DNA synthesis by cisplatin was largely unaffected by forced expression of TS (Figure 3B). An annexin V binding assay also revealed that the frequency of apoptosis was markedly increased by pemetrexed in a concentration-dependent manner in Mock cells, whereas pemetrexed had little effect on apoptosis in cells overexpressing TS (Figure 4A). To confirm that this attenuation of pemetrexed-induced apoptosis in TS-overexpressing cells was due to the forced expression of TS, we depleted the TS-overexpressing cells of TS by transfection with the TS siRNA and then examined the effect of pemetrexed on apoptosis. Downregulation of TS expression restored the sensitivity of these cells to the proapoptotic effect of pemetrexed. In contrast to pemetrexed, cisplatin increased the proportion of apoptotic cells among Mock and TS-overexpressing cells to similar extents (Figure 4C). These data thus suggested that the effects of pemetrexed on DNA synthesis and apoptosis are inversely related to the level of TS expression.

### Effects of pemetrexed on the growth of TS-overexpressing NSCLC cells *in vivo*

**Figure 5:**
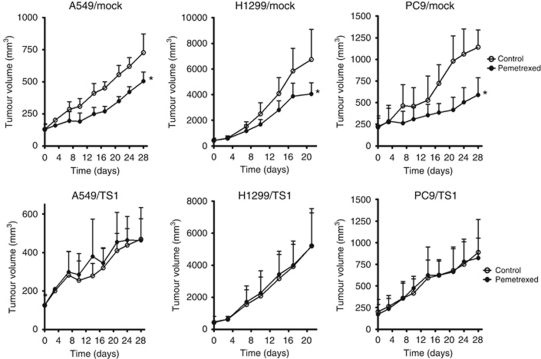
Effect of pemetrexed on the growth of TS-overexpressing NSCLC cells *in vivo*. Nude mice with tumour xenografts established by subcutaneous implantation of tumour fragments derived from the indicated NSCLC cell lines were treated with vehicle (control) or pemetrexed (100 mg kg^−1^, intraperitoneal) on days 1, 8, 15, and 22. Tumour volume was determined at the indicated times after the onset of treatment. Data are means±s.e.m. of values from eight mice per group. ^*^P<0.05 for pemetrexed *vs* the corresponding value for vehicle (Student's two-tailed *t*-test).

We next investigated whether TS-overexpressing NSCLC cell lines might exhibit resistance to pemetrexed treatment in xenograft models. When their tumours became palpable, athymic nude mice were divided into two groups and treated with vehicle or pemetrexed for 3–4 weeks. Although pemetrexed significantly inhibited the growth of tumours formed by Mock cells of the A549, H1299, or PC9 lines, it did not exhibit such an effect with tumours formed by the corresponding TS-overexpressing cells (Figure 5). These data thus suggested that the antitumour effect of pemetrexed is suppressed by TS overexpression in NSCLC cells, consistent with our results obtained *in vitro*.

### TS expression in tumours of NSCLC patients treated with pemetrexed

**Figure 6:**
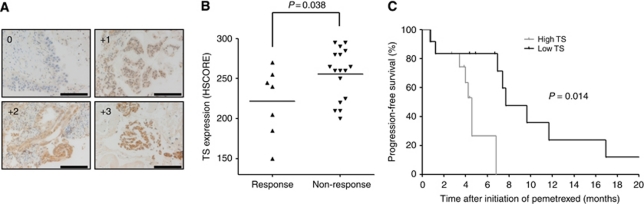
Relation of TS expression level to tumour response in NSCLC patients treated with pemetrexed and either carboplatin or cisplatin. (**A**) Representative sections of carcinomas including cells with the indicated intensities of TS immunostaining. Scale bars, 125 *μ*m. (**B**) TS expression level (HSCORE) for the clinical specimens of 24 patients classified according to tumour response (response=CR or PR, *n*=7; non-response=SD or PD, *n*=17). Horizontal lines indicate mean values. The *P* value was determined by Student's two-tailed *t* test. (**C**) Progression-free survival of the NSCLC patients according to the expression level of TS in tumour specimens. The *P*-value was determined with the log-rank test.

To evaluate the relation between the level of TS expression in NSCLC tumours and the clinical response to pemetrexed, we performed semiquantitative immunohistochemical analysis on tumour biopsy specimens from 24 patients with advanced NSCLC treated with pemetrexed combined with platinum agents (Figure 6A). The characteristics of the patients are shown in Table 2. Tumours were categorised as either responding (CR or PR) or non-responding (SD or PD). The level of TS expression for non-responding groups was significantly (*P*=0.038) higher than that for responding groups (Figure 6B). We next carried out ROC curve analysis to establish the optimal cutoff value for the HSCORE of TS expression level, yielding a value of 257.5. Patients with a low level of TS expression (HSCORE<257.5) had a significantly longer progression-free survival (*P*=0.014) than did those with a high level (HSCORE⩾257.5) (Figure 6C). These data thus suggested that TS expression level in advanced NSCLC tumours is inversely correlated with the response to pemetrexed.

## Discussion

In this study, we have investigated the effects of TS overexpression on the sensitivity of NSCLC cells to pemetrexed. Pemetrexed-resistant lung cancer cell lines established by stepwise exposure to increasing concentrations of pemetrexed were recently shown to contain increased amounts of TS mRNA compared with parental cells (Ozasa *et al*, 2010). Other previous studies have also found that sensitivity to pemetrexed is inversely related to the level of TS expression in cancer cell lines (Sigmond *et al*, 2003; Giovannetti *et al*, 2008). These observations have suggested that TS gene expression is associated with resistance to pemetrexed, but the mechanism by which a high level of TS expression might result in a reduced sensitivity to pemetrexed has remained unclear. Thymidylate synthase has a central role in the biosynthesis of thymidylate, an essential precursor for DNA synthesis (Carreras and Santi, 1995). We have previously shown that TS expression level differs among lung cancer cell lines, and that RNA interference-mediated depletion of TS in such cell lines resulted in growth suppression through inhibition of DNA synthesis and induction of apoptosis in a manner independent of the original level of TS activity (Takezawa *et al*, 2010). Pemetrexed exerts its cytotoxic effects through inhibition of multiple DNA synthesis-related enzymes including TS. We have now shown that pemetrexed inhibited DNA synthesis and induced apoptosis in NSCLC cell lines; however, it failed to induce such effects in the corresponding cells engineered to overexpress TS. Forced expression of TS also abolished the antitumour effect of pemetrexed in xenograft models. Our data suggest that pemetrexed did not fully inhibit TS activity in TS-overexpressing cells, given that DNA synthesis remained active after pemetrexed exposure. They further suggest that the observed reduction in the sensitivity of TS-overexpressing cells to pemetrexed may result from sustained activity of TS in the presence of the drug. We also examined the possible effect of pemetrexed on the expression levels of apoptosis-related molecules in both Mock and TS-overexpressing cells, but we found that pemetrexed did not substantially alter the abundance of such proteins including that of XIAP (data not shown), which we previously identified as having a key role in TS depletion-induced apoptosis in NSCLC cells (Takezawa *et al*, 2010). The precise mechanism by which pemetrexed induces apoptosis thus remains to be determined.

Previous studies have examined the possible relation between TS expression level and the response to pemetrexed in cancer patients (Gomez *et al*, 2006; Righi *et al*, 2010; Uramoto *et al*, 2010). In a phase II trial of pemetrexed monotherapy for advanced breast cancer (Gomez *et al*, 2006), 61 patients were treated with pemetrexed and evaluable for response. This study revealed a potential association between a high level of TS mRNA and a poor response to pemetrexed treatment. Another study evaluated TS expression level immunohistochemically by means of the HSCORE system in 60 patients with malignant mesothelioma treated either with the combination of pemetrexed and platinum, or with pemetrexed alone (Righi *et al*, 2010). A significant inverse correlation was found between TS expression level and time to progression, or overall survival. Finally, no significant correlation between the abundance of TS mRNA and clinical outcome was apparent for five NSCLC patients treated with the combination of pemetrexed and platinum, or with pemetrexed alone (Uramoto *et al*, 2010), although the small sample number precluded any definitive conclusion. In this study, we found that a high level of TS expression in human NSCLC tumours was significantly associated with a reduced tumour response and a shorter progression-free survival in 24 patients treated with pemetrexed combined with platinum agents, consistent with the previous studies of patients with breast cancer or malignant mesothelioma (Gomez *et al*, 2006; Righi *et al*, 2010). Given that the anticancer effects of cisplatin were independent of TS expression level in NSCLC cell lines, the relation between TS expression level and clinical outcome observed in our clinical analysis likely reflects the effect of pemetrexed. We recently evaluated the abundance of TS in NSCLC tumours of patients treated with carboplatin and paclitaxel, and neither a prognostic nor predictive role was identified for TS expression level in these patients (Takeda *et al*, 2010). Together with such observations, our present results suggest that assessment of baseline TS expression may be of predictive value in evaluation of chemosensitivity to pemetrexed in NSCLC. Although we cannot exclude a contribution of factors other than TS expression level to pemetrexed chemosensitivity, our preclinical and clinical data provide a rationale for the potential use of TS expression level as a predictive biomarker for response to pemetrexed or pemetrexed-based chemotherapy in patients with NSCLC. Further investigation is needed with a larger cohort of patients or in prospective studies to confirm this conclusion.

## Figures and Tables

**Table 1:** Median inhibitory concentrations (*μ*M) for the antiproliferative effects of chemotherapeutic agents in TS-overexpressing NSCLC cells *in vitro*

**Cell line**	**Pemetrexed**	**Cisplatin**	**Docetaxel**
A549/Mock	0.07	2.62	0.12
A549/TS1	0.38	2.37	0.12
A549/TS2	0.44	2.21	0.13
H1299/Mock	0.08	2.93	0.32
H1299/TS1	0.22	2.98	0.30
H1299/TS2	0.22	2.90	0.30
PC9/Mock	0.03	0.72	0.18
PC9/TS1	0.11	0.72	0.18
PC9/TS2	0.10	0.65	0.17

Abbreviations: NSCLC=non-small cell lung cancer; TS=thymidylate synthase.

**Table 2:** Patient characteristics

**Characteristic**	
*Sex*	
Male	17
Female	7
Mean (range) age (years)	66 (38–85)
	
*Chemotherapy*	
Carboplatin+pemetrexed	23
Cisplatin+pemetrexed	1
	
*Tumor histology*	
Adenocarcinoma	21
Squamous cell	1
Other	2
	
*Disease stage*	
IIIB	7
IV	17
	
*Tumor response*	
CR+PR	7
SD	13
PD	4

Abbreviations: CR=complete response; PD=progressive disease; PR=partial response; SD=stable disease.
